# Malignant Peritoneum Mesothelioma with Hepatic Involvement: A Single Institution Experience in 5 Patients and Review of the Literature

**DOI:** 10.1155/2016/6242149

**Published:** 2016-03-16

**Authors:** Shan-shan Su, Guo-qi Zheng, Ya-gang Liu, Yue-feng Chen, Zhao-wei Song, Shu-jing Yu, Ning-ning Sun, Yu-xin Yang

**Affiliations:** Department of Gastroenterology, Cangzhou Central Hospital, Cangzhou, Hebei 061001, China

## Abstract

Malignant peritoneal mesothelioma with invasion of the liver is an invariably fatal disease. We aimed to clarify the characteristics of malignant peritoneal mesothelioma cases with liver involvement. The clinical presentation, computed tomography images, and immunohistochemical and histopathological features of 5 patients with malignant peritoneal mesothelioma and liver involvement were evaluated. The diagnosis was established by imaging and immune profiles of the tumours. A review of 8 cases with primary or invading malignant mesothelioma in liver is presented. All 5 mesothelioma cases were asbestos-related. CT images of malignant peritoneal mesothelioma with the liver involvement typically showed that the lesion grew inside the liver along the capsule and was possibly accompanied by capsule breakthrough and extrahepatic infiltration. The tumours exhibited a common epithelioid appearance in all 5 patients and most cases revealed positive Cal, CK, and MC with negative CEA and HeP. Different from our findings, the review of literature revealed that most malignant mesothelioma of liver was due to primary intrahepatic malignant mesothelioma. Finally, we concluded that the diagnosis of malignant peritoneal mesothelioma cases with liver invasion is reliably achieved by the history of asbestos exposure, the characteristic CT imaging, and immune profiles of the tumours.

## 1. Background

Malignant mesothelioma is a tumour of the lining of the lung and chest cavity or the lining of the abdomen and is typically related to asbestos exposure [1–4]. Next to malignant pleural mesothelioma, malignant peritoneal mesothelioma (MPM) is the second most common type of malignant mesothelioma. Approximately 35% of all mesotheliomas arise solely from the peritoneum [5]. MPM has been reported to invade adjacent visceral structures, such as the liver, spleen, or pelvic organs [6]. To date, there are few reports of primary or secondary MPM in the liver, and each such report presents a single case [7–14]. In this study, our report identifies 5 patients with MPM invading the liver to clarify the characteristics of MPM invading the liver and to review cases with primary or invading MPM in liver.

## 2. Patients and Methods

The study was approved by the ethics committee of Central Hospital of Cangzhou City, Cangzhou, Hebei, China (reference number: 2012-012-01).

Five patients treated at our hospital with the diagnosis of MPM invading the liver from May 2011 to September 2014 were evaluated. All the patients received thoracic, abdominal, and pelvic computed tomography (CT) scans as well as enhanced CT, which identified malignant mesothelioma of the peritoneum without other primary tumours. Radiologists (who had more than 10 years of experience in abdominal imaging) in our hospital had interpreted the images of the 5 patients. Three radiologists independently interpreted the CT images, and discrepancies in the CT findings were resolved by a consensus opinion of at least two interpreters. The histological diagnosis of eligible patients was in accordance with the Guidelines for Pathologic Diagnosis of Malignant Mesothelioma in 2012 from the International Mesothelioma Interest Group [15].

## 3. Results

### 3.1. Characteristics of Cases

The principal characteristics of the cases are summarised in Table 1.

The 5 patients included 1 male and 4 females. The mean age at the time of diagnosis was 57.6 (range: 45–69) years. All 5 cases were positive for asbestos exposure and not comorbidity which included malignant pleural mesothelioma. All patients complained of abdominal distention and pain; 4 cases displayed massive ascites, and 1 case had no ascites. One case presented with ovarian mass. The average serum cancer antigen 125 (CA125) level (normal value < 35.0 U/mL) was 263.5 U/mL (range 36.6–725.3 U/mL), and the cholestatic parameters, transaminases, AFP, CA19-9, and CEA, were within normal range in the 5 cases.

### 3.2. CT Manifestations

**Figure 1:**
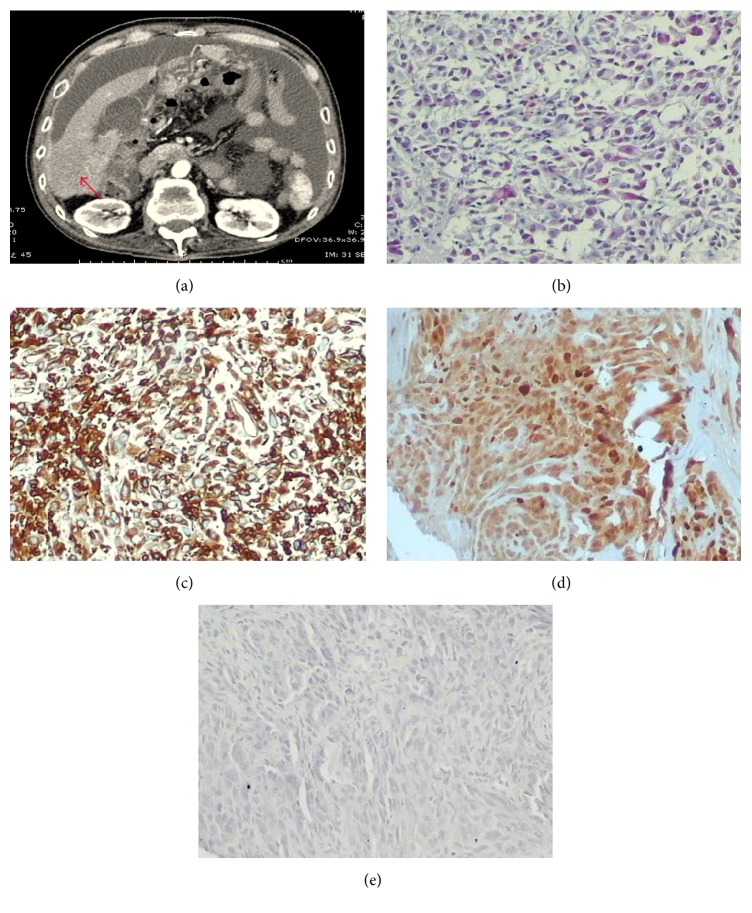
Abdominal CT and cytohistological features on H&E-stained specimens or immunohistochemical stains for case 1. (a) Abdominal CT demonstrated a mass in the hepatic flexure of colon with invasion into the liver (red arrows). (b) Cytohistological features of the greater omentum biopsy on H&E-stained specimens. Immunohistochemical stains showed that the tumour cells were positive for cytokeratin (c) and calretinin (d) but negative for carcinoembryonic antigen (e).

**Figure 2:**
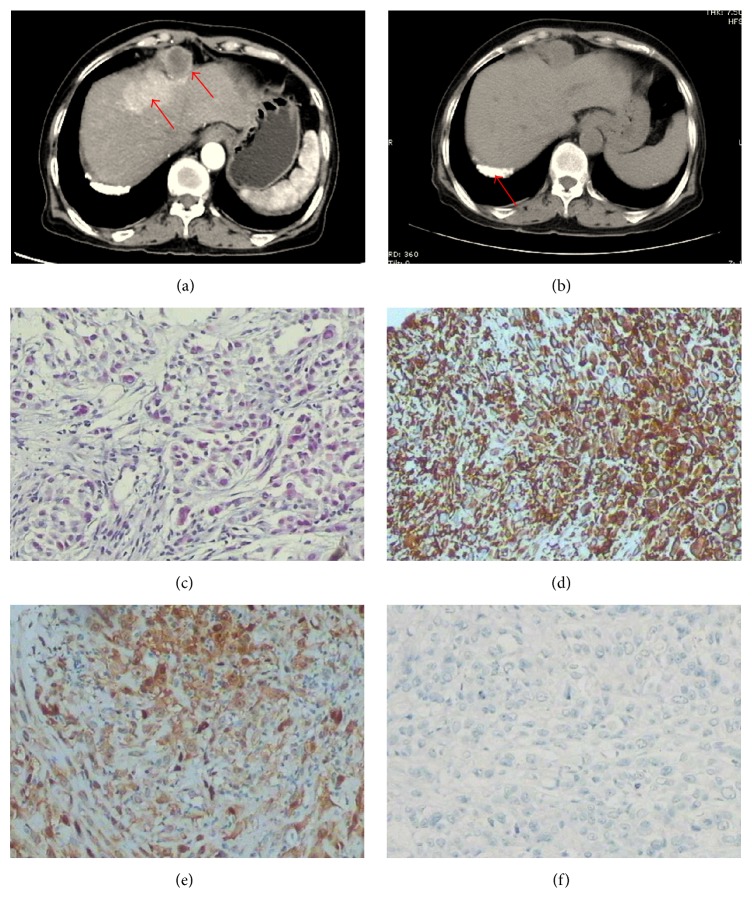
Abdominal CT and cytohistological features on H&E-stained specimens or immunohistochemical stains for case 2. (a) Abdominal CT demonstrated hepatic nodule in the VI segment of the right lobe of the liver and there were nodules in the soft tissue anterior to the liver (red arrows). (b) Abdominal CT demonstrated pleural plaque (red arrows). (c) Cytohistological features of the nodules biopsy in the soft tissue anterior to the liver on H&E-stained specimens. Immunohistochemical stains showed that the tumour cells were positive for mesothelial cell antibody (d) and calretinin (e) but negative for carcinoembryonic antigen (f).

**Figure 3:**
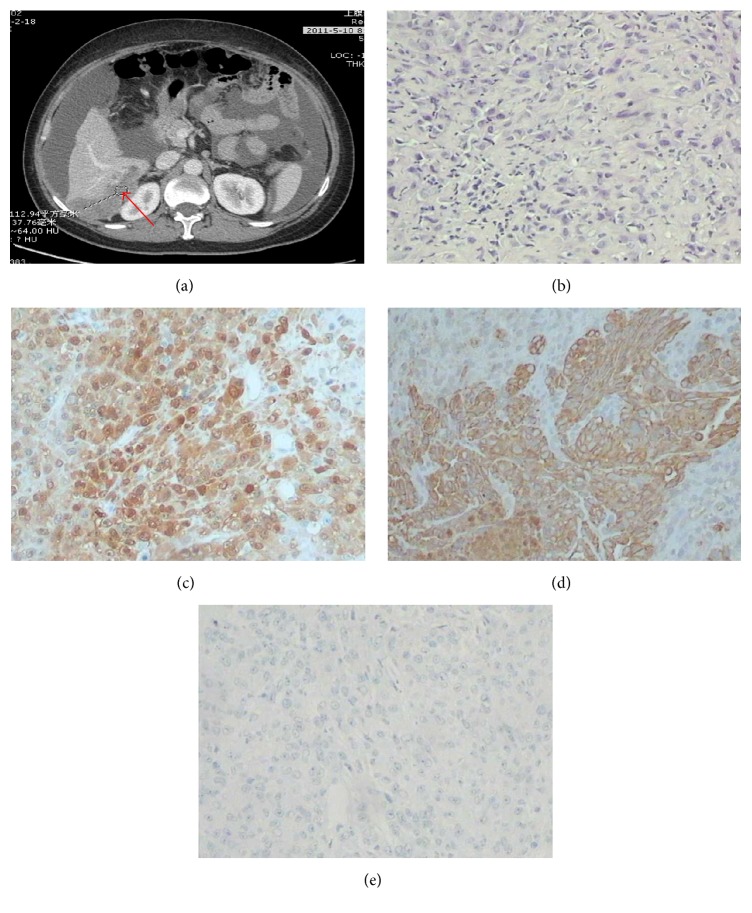
Abdominal CT and cytohistological features on H&E-stained specimens or immunohistochemical stains for case 3. (a) Abdominal CT demonstrated that the greater omentum and peritoneum around the liver were thickened accompanying mass shade, with infiltration of the posterior segment of right liver (red arrows). (b) Cytohistological features of the peritoneum biopsy on H&E-stained specimens. Immunohistochemical stains showed that the tumour cells were positive for calretinin (c) and cytokeratin (d) but negative for carcinoembryonic antigen (e).

**Figure 4:**
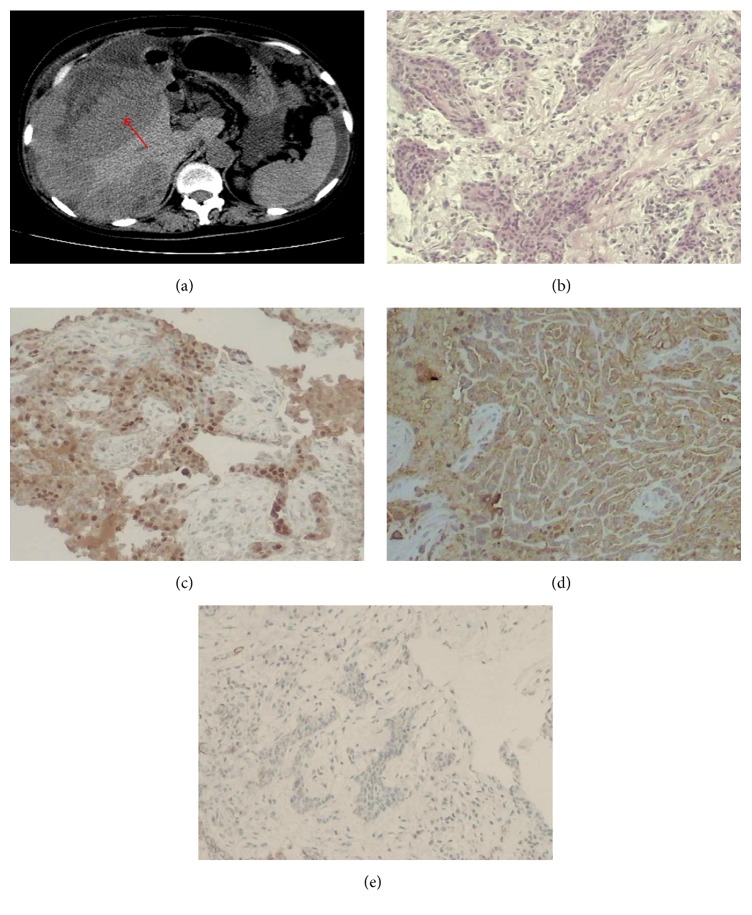
Abdominal CT and cytohistological features on H&E-stained specimens or immunohistochemical stains for case 4. (a) Abdominal CT demonstrated a huge mass with heterogeneous density in the right flank, the peritoneum, and omentum thickening accompanied by multiple masses infiltrating the inferior pole of right liver (red arrows). (b) Cytohistological features of the peritoneum biopsy on H&E-stained specimens. Immunohistochemical stains showed that the tumour cells were positive for calretinin (c) and epithelial membrane antigen (d) but negative for carcinoembryonic antigen (e).

**Figure 5:**
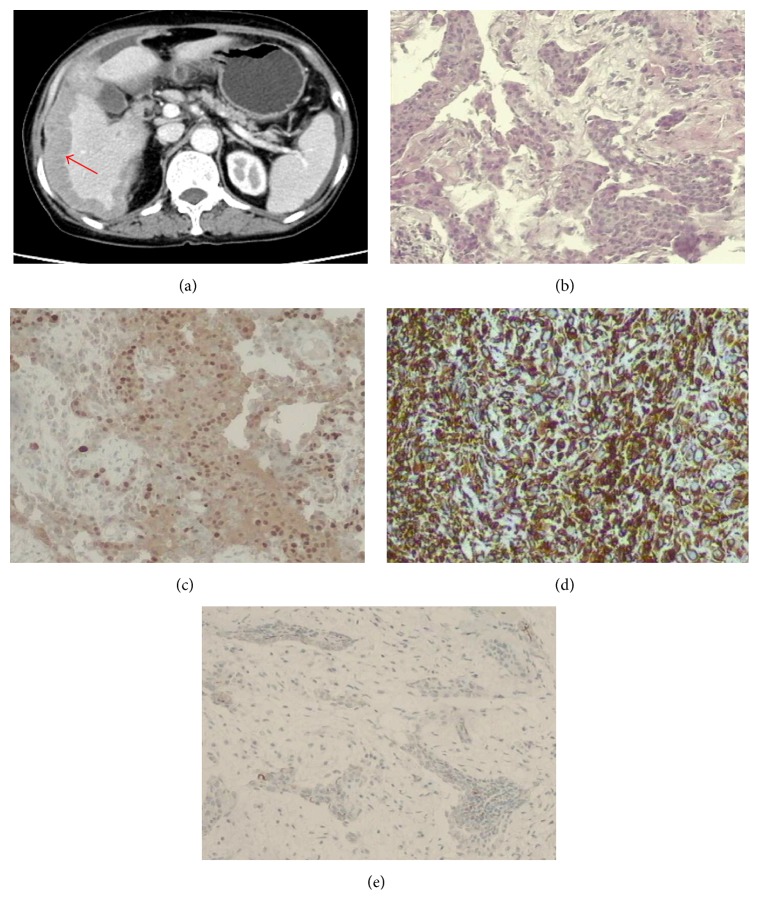
Abdominal CT and cytohistological features on H&E-stained specimens or immunohistochemical stains for case 5. (a) Abdominal enhanced CT revealed nonuniform thickening of peritoneum around the liver with associated invasion in liver (red arrows). (b) Cytohistological features of the peritoneum biopsy on H&E-stained specimens. Immunohistochemical stains showed that the tumour cells were positive for calretinin (c) and mesothelial cell antibody (d) but negative for carcinoembryonic antigen (e).

Abdominal enhanced computed tomography (CT) for case 1 demonstrated a mass in the hepatic flexure of colon invading the liver and lung with peritoneal thickening, and the mass was enhanced significantly in enhanced CT (Figure 1). For case 2, abdominal enhanced CT revealed a hepatic nodule in the VI segment of the right lobe. There were multiple nodules in the soft tissue anterior to the liver. Peripheral enhancement of the hepatic nodule was observed on enhanced CT (Figure 2). Furthermore, there was obstruction of biliary tract and infiltration of lymph nodes in this case. Abdominal enhanced CT for case 3 demonstrated that the greater omentum and peritoneum around the liver were thickened and had an accompanying mass, and there was infiltration of the posterior segment of the right liver and metastases in lymph nodes in the cardiophrenic angle (Figure 3). Abdominal enhanced CT for case 4 demonstrated a huge mass approximately 16 cm × 10 cm × 21 cm with heterogeneous density in the right flank and with peritoneum and omentum thickening accompanied by multiple masses infiltrating the inferior pole of the right liver (VI segment) (Figure 4). Finally, for case 5, abdominal enhanced CT revealed nonuniform thickening of peritoneum around the liver with associated invasion in liver and metastases in the cardiophrenic angle lymph nodes and hepatic portal (Figure 5). Pleural plaques were found in cases 2, 3, and 5. Cases 1, 2, 3, and 5 were localised MPM, whereas case 4 was diffuse type.

### 3.3. Pathologic Manifestations

Pathologic examination of the 5 cases proved that the liver was involved. Liver involvement displayed a nodular and infiltrative pattern. The tumours exhibited a common epithelioid appearance in all 5 cases. Each tumour was examined by immunohistochemical markers according to the potential differential diagnosis. Table 1 summarises the immunohistochemical panel. The positivity rates for each marker were 5/5 (positive cases/analysed cases) for calretinin (Cal); 3/5 for mesothelin (MC); 4/5 for cytokeratin (CK); 2/2 for epithelial membrane antigen (EMA); 0/5 for hepatocyte (HeP); and 0/5 for carcinoembryonic antigen (CEA).

### 3.4. Outcomes

All 5 cases had follow-up. Only case 5 remains alive at this time, and the average survival time for the other 4 cases from initial diagnosis was 6.5 months.

## 4. Discussion 

MPM is a rare malignancy, with an incidence of one case in a population of 4-5 million [16], and it has a poor clinical course, with death occurring within 2 years of diagnosis in most patients. Similar to malignant pleural mesothelioma, histology, clinical experience, radiological imaging, electron microscopy, and immunohistochemistry are crucial in making an accurate diagnosis [17]. MPM cases with liver invasion are much rarer, and it is difficult to distinguish them from liver cancer or localised tuberculous peritonitis near the liver.

### 4.1. Aetiology

Asbestos exposure has been shown to be an aetiology of MPM [18–20]. In the absence of heavy exposure, asbestos fibers mostly accumulate in the lungs where some of them may induce the occurrence of lung cancer while others may migrate to the lymph nodes/lymphatics as well as to the pleura and eventually to the peritoneum, which promote malignant mesothelioma development [17]. Our previous study reported that 93.2% of 162 MPM patients had a history of asbestos exposure, and most of this exposure was chrysotile exposure [21]. In this study, we found that all 5 cases had experienced chrysotile exposure. The role of chrysotile in MPM continues to be debated, though the role of amphibole asbestos in malignant mesothelioma pathogenesis is well established. There is a general agreement that amphibole asbestos, particularly crocidolite, is a much more potent carcinogen that causes MPM compared with forms of serpentine asbestos, such as chrysotile [16, 22–24]. However, it is accepted that chrysotile can cause lung cancer in humans and malignant mesothelioma in rats. Some authors have proposed that even if chrysotile is less potent than amphiboles, it remains a known carcinogen and accounts for approximately 95% of the asbestos used worldwide. Therefore, chrysotile might be the main cause of MPM.

Several reports have reported secondary liver involvement by MPM or primary hepatic mesothelioma. MPM invading the liver was reported in 1 case, and primary hepatic mesothelioma was reported in 7 patients [7–14] (Table 2). However, only 1 of these 8 patients had a history of asbestos exposure, which indicated that asbestos exposure was more common in patients with MPM in our region.

It has been reported that MPM is more prevalent in males [25] and that it may be associated with more prolonged, heavy asbestos exposure than pleural MM [26]. However, our 5 cases included 4 women and only 1 man, which might result from the fact that, in this region, females were the main producers of hand-spun asbestos in the 1970s [27]. Most MPM patients were those who had the greatest cumulative asbestos exposure. In this region, MPM is not as rare as in the reported literature, which likely results from the poor protective measures implemented during hand spinning in asbestos processing.

### 4.2. Presentation

Because symptoms and clinical course are usually nonspecific during the early presentation of MPM, a suspected diagnosis of carcinomatosis of unknown origin is occasionally given to patients. All 5 patients complained of abdominal distention and pain, and 1 case presented with an ovarian mass, which demonstrates that the clinical presentation of MPM cases with liver involvement is not specific.

It is known that MPM frequently expresses CA125 [28–30]. All our 5 cases were found to have elevated serum CA125, but the serum AFP, CA 19-9, and CEA levels were within the normal ranges, which is consistent with the report of Kebapci et al. [30]. This profile of tumour markers may strongly suggest mesothelioma rather than other types of cancer.

### 4.3. Imaging Performance

CT findings are useful for detection and staging of peritoneal masses. For malignant pleural mesothelioma, the chest CT mainly shows a nodular or cyclic thickening of the pleura, the mediastinal pleural involvement with different degrees of pleural effusion. The enhanced CT images can be characterised by the multiple reinforcement nodules on the pleura [31]. For MPM, the CT images manifest with diffuse nodules and plaques which tend to envelop the bowel viscera or with a large tumour mass that is usually in the upper abdomen, and there may be discrete nodules scattered throughout the peritoneum [29]. Otherwise, omental involvement in MPM ranges from heterogeneity of the fat with streaky density to the classic “omental cake” appearance [29].

Pleural plaques are found in approximately 50% of all patients with MPM [32]. In our study, 3 of the 5 cases contained pleural plaques, which indicated that the pleural plaque might be a diagnostic marker for MPM (Figure 2(b)). Abdominal CT scans revealed intrahepatic masses continuous to the peritoneal thickening or nodules, which suggested that tumours arise from both parietal and visceral peritoneum. The CT images of MPM invading the liver typically showed that the lesion grew inside the liver along the capsule and was accompanied by capsular breakthrough and extrahepatic infiltration. Peritoneum around the liver and/or omentum was typically thickened, with the masses accompanied by a single liver nodule. Diffuse parietal peritoneum with clearly irregular thickening and nodular tumour implants on the undersurface of the right diaphragm can indent the liver surface. In case 5, this phenomenon was observed, and the peritoneum was thickened to 2.6 cm. Most MPM entailed direct invasion to adjacent tissues or organs rather than hematogenous or lymphatic spread as distant metastasis. MPM can be divided into diffuse malignant peritoneal mesothelioma (DMPM) and localised malignant peritoneal mesothelioma (LMPM), with DMPM accounting for approximately 82.1% of all MPM cases [1]. However, 4 of the cases in this report were LMPM, whereas only 1 case was DMPM, which may indicate that LMPM more commonly has liver involvement.

The review of the literature revealed some case of malignant mesothelioma in liver due to primary intrahepatic malignant mesothelioma [7–14]. However, all the cases in our study were malignant mesothelioma of the peritoneum with subsequent liver invasion. We asked experts in radiology and surgery in our hospital to interpret the images provided in the literature, and they found that these lesions were also subcapsular and possibly continuous to the peritoneum in all 7 additional patients.

Sasaki et al. speculated that primary hepatic mesothelioma might originate from mesothelial cells of Glisson's capsule which subsequently invade the liver [9]. In contrast to this speculation, we do not believe primary hepatic mesothelioma with high morbidity. Glisson's capsule consists of collagen fibers, including type I and type III collagen, fibroblast cells, and small blood vessels. There are no mesothelial cells in Glisson's capsule. Therefore, if the tumour originates from mesothelial cells, it would be MPM rather than primary hepatic mesothelioma. We know that MPM spreads along the parietal and visceral peritoneal surfaces and encases the peritoneal cavity and intraperitoneal organs [6]. In progressive disease, the tumour may infiltrate the viscera. Hepatic invasion or intrahepatic metastasis may occur in MPM. Here, the presence of peritoneal involvement in all 5 patients suggests that these cases were a primary malignant tumour of peritoneum with hepatic invasion and were thus not of primary liver origin. Because mesothelial cells are not present in the liver under normal physiological conditions [9], the present tumour might originate from mesothelial cells of the peritoneum, which subsequently invaded the liver.

### 4.4. Histopathology and Immunohistochemistry

CT findings that suggest MPM are not sufficient to establish a definitive diagnosis of mesothelioma. Peritoneal biopsy could confirm the diagnosis of MPM. Immunohistochemical panels are absolutely essential for the pathological and differential diagnosis of MPM [33, 34]. Here, MPM was diagnosed by considering tumour localisation and microscopic and immunohistochemical findings. Histological and immunohistochemical findings also led to the diagnosis of intrahepatic mesothelioma infiltration. Figures 1–5 show representative cases with common mesothelioma morphology, liver involvement, and the profile of positive and negative immune markers.

Histologically, MPM conforms to one of three patterns: epithelial (the most common type), sarcomatoid, or biphasic (mixture of epithelioid and sarcomatoid) types [26]. The tumours exhibited a common epithelioid appearance in all 5 patients in our study. The review of the previous literature showed that tumours had an epithelioid pattern in 7 patients and a biphasic pattern in 1 patient, which indicated that the epithelioid pattern is the most common histologic appearance of MPM invading liver.

Because there is no single absolute marker for mesothelioma [35, 36], two or more positive immunohistochemical mesothelial markers combined with negative epithelial (adenocarcinoma) markers are recommended for the diagnosis of mesothelioma [37, 38]. Cal, CK, and MC are proposed as positive markers for mesothelioma because they are commonly expressed in mesotheliomas but not in carcinomas. MPM is characterised by membranous EMA positivity as well, but this marker does not discriminate from adenocarcinoma [39]. Negative CEA is helpful in distinguishing mesothelioma from adenocarcinoma [39]. Most cases in this study revealed positive Cal, CK, and MC with negative CEA and HeP, which indicated mesothelioma rather than a different type of cancer.

### 4.5. Differential Diagnosis

MPM is difficult to distinguish from liver cancer or localised tuberculous peritonitis near the liver.

As noted above, CT imaging of MPM invading the liver usually shows a lesion growing inside the liver accompanied by capsular breakthrough and extrahepatic infiltration. Peritoneum around the liver and/or omentum was usually thickened with masses present.

CT images of cholangiocellular carcinoma and hepatocellular carcinoma show a mass with an intrahepatic location, usually with an internal mosaic pattern, a pseudocapsule around the nodule, the presence of fat, vascular invasion, and satellite nodules. There are rare tumours breaking through the peritoneal cavity, and not lesions, which suggest primary tumours in other organs. In addition, portal vein tumour thrombosis is a well-known complication. These types of carcinoma can induce distant metastasis by hematogenous spread. The immunohistochemical findings usually indicate that these cancers are positive for AFP and HeP and negative for Cal and MC.

**Figure 6:**
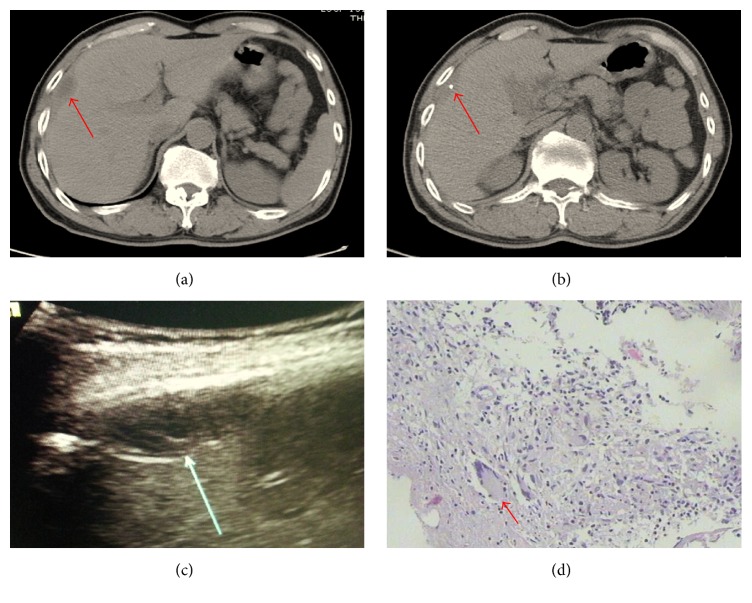
Abdominal CT, ultrasonography, and cytohistological features on H&E-stained specimens for localised tuberculous peritonitis. (a), (b) Abdominal CT and (c) ultrasonography demonstrated a hepatic lesion (arrows). (d) Cytohistological features of the liver biopsy on H&E-stained specimens display the presence of Langhans cells (red arrows).

MPM may present with abdominal distention, pain, and ascites. It is difficult to distinguish tuberculous peritonitis when the lesion is near the liver from MPM invading the liver. Most patients with tuberculous peritonitis have a history of tuberculosis and have symptoms of mild fever and night sweats. We compared the CT scans of MPM invading the liver with a case of localised tuberculous peritonitis near the liver. As the images show, the lesion was near the liver capsule with liquid accumulation and central calcification but no intrahepatic lesion or peritoneal breakthrough in localised tuberculous peritonitis (Figure 6). The histological morphology of localised tuberculous peritonitis may show the presence of Langerhans cells (Figure 6), and the immunohistochemical stains usually show the cells to be CD68 positive.

### 4.6. Treatment and Results

For small and limited tumours, the mass and the involved organs should be completely resected. The nonsurgical therapeutic options in MPM are very limited. Radiation is only feasible for local tumour control, and multimodal treatments with chemotherapy (e.g., pemetrexed) can achieve partial remission in most cases of MPM [40]. In our study, intravenous pemetrexed and cisplatin were used for case 5, the patient who had the longest survival time. The mean survival time was 6.5 months (range: 5–8 months) for the other 4 patients.

## 5. Conclusion

In conclusion, there are likely rare cases of primary hepatic mesothelioma but more cases of MPM with hepatic invasion. MPM invading the liver should be included in the differential diagnosis of hepatic tumours or other diseases that present similar CT results. Patients who have the following characteristics may have MPM with hepatic invasion: (a) history of asbestos exposure; (b) presence of pleural plaque in CT image; (c) clearly thickened peritoneum in CT image; (d) subcapsular liver lesions that are continuous with the peritoneum in CT image; (e) thickened omentum and peritoneum around liver and/or with presence of masses in CT image; and/or (f) positive for Cal, CK, and MC but negative for CEA and HeP in immunohistochemical assays. Careful CT imaging and histological and immunohistochemical analyses are required to reach a final diagnosis. Furthermore, the accumulation of additional cases similar to the current cases is necessary to characterise the features of MPM with liver involvement, including its biological behaviour and prognosis.

## Figures and Tables

**Table 1:** Clinical features and outcomes of the patients.

Case	Age	Sex	History of asbestos exposure	Clinical manifestation	Physical examination	Ascites	Positive immunohistochemical stains	Negative immunohistochemical stains	Treatment	Overall survival (months)
1	57	M	+	Abdominal distension	Abdominal tenderness	Massive ascites	Cal, MC, CK	CEA, HeP	Intraperitoneal cisplatin	5

2	69	F	+	Abdominal pain and distension	Abdominal tenderness	No ascites	Cal, MC, CK	CEA, HeP	Intraperitoneal cisplatin	6

3	51	F	+	Abdominal distension	Fullness in the flanks	Massive ascites	Cal, CK, EMA	CEA, HeP, MC	Intravenous and intraperitoneal cisplatin	7

4	45	F	+	Abdominal mass	A mass in the right abdomen	Massive ascites	Cal, EMA	CEA, HeP, MC, CK	Oophorectomy, omentectomy, and adjuvant chemotherapy	8

5	66	F	+	Abdominal distension	Abdominal tenderness	Massive ascites	Cal, MC, CK	CEA, HeP	Intravenous pemetrexed and cisplatin	/

Note: M: male; F: female; +: positive; Cal: calretinin; MC: mesothelin; CK: cytokeratin; HeP: hepatocyte; CEA: carcinoembryonic antigen; EMA: epithelial membrane antigen; /: alive up to now.

**Table 2:** Outlines of reported cases of MPM invading liver and primary hepatic mesothelioma.

Year of publication and reference	Age/sex	History of asbestos exposure	Pathological pattern	Imaging or operation performance	Diagnosis
2013 [7]	68/F	−	Epithelial type	An intrahepatic tumor in the right lobe of the liver with cervical, axillary, and abdominal para-aortic lymph node swellings	Primary hepatic mesothelioma

2011 [8]	58/F	−	Epithelial type	An intrahepatic nodule in the posterior segment, with peritoneal thickening, multiple nodular lesions in the pelvic cavity, and para-aortic and intra-abdominal lymphadenopathy	MPM invading liver

2009 [9]	66/M	+	Biphasic type	A hepatic nodule in the S8 segment of the liver just under the diaphragm	Primary hepatic mesothelioma

2009 [10]	62/M	−	Epithelial type	An mass in the right lobe of the liver near the right adrenal gland	Primary hepatic mesothelioma

2008 [11]	53/M	−	Epithelial type	A hepatic mass at hepatic dome	Primary hepatic mesothelioma

2006 [12]	62/M	−	Epithelial type	A mass with cystic areas in the right liver lobe near the right adrenal gland	Primary hepatic mesothelioma

2003 [13]	54/F	−	Epithelial type	A heterogeneous mass in the right lobe of the liver	Primary hepatic mesothelioma

2002 [14]	64/M	−	Epithelial type	A tumor nodule in the right hepatic lobe	Primary hepatic mesothelioma

Note: M: male; F: female; +: positive; −: negative.

## Abbreviations

MPM: Malignant peritoneal mesothelioma
CT: Computed tomography
Cal: Calretinin
MC: Mesothelin
HeP: Hepatocyte
CEA: Carcinoembryonic antigen
Vim: Vimentin
CK: Cytokeratin.

## References

[1] Remon J., Lianes P., Martínez S., Velasco M., Querol R., Zanui M. (2013). Malignant mesothelioma: new insights into a rare disease. *Cancer Treatment Reviews*.

[2] Raja S., Murthy S. C., Mason D. P. (2011). Malignant pleural mesothelioma. *Current Oncology Reports*.

[3] Chapman E. A., Thomas P. S., Yates D. H. (2010). Breath analysis in asbestos-related disorders: a review of the literature and potential future applications. *Journal of Breath Research*.

[4] Pedata P., Feola D., Laieta M. T., Garzillo E. M. (2011). Peritoneal mesothelioma: description of a case and review of literature. *International Journal of Immunopathology and Pharmacology*.

[5] Robinson B. W. S., Lake R. A. (2005). Advances in malignant mesothelioma. *The New England Journal of Medicine*.

[6] Levy A. D., Arnáiz J., Shaw J. C., Sobin L. H. (2008). From the archives of the AFIP Primary peritoneal tumors: imaging features with pathologic correlation. *Radiographics*.

[7] Inagaki N., Kibata K., Tamaki T., Shimizu T., Nomura S. (2013). Primary intrahepatic malignant mesothelioma with multiple lymphadenopathies due to non-tuberculous mycobacteria: a case report and review of the literature. *Oncology Letters*.

[8] Nagata S., Tomoeda M., Kubo C. (2011). Malignant mesothelioma of the peritoneum invading the liver and mimicking metastatic carcinoma: a case report. *Pathology Research and Practice*.

[9] Sasaki M., Araki I., Yasui T. (2009). Primary localized malignant biphasic mesothelioma of the liver in a patient with asbestosis. *World Journal of Gastroenterology*.

[10] Buchholz B. M., Gütgemann I., Fischer H.-P. (2009). Lymph node dissection in primary intrahepatic malignant mesothelioma: case report and implications for diagnosis and therapy. *Langenbeck's Archives of Surgery*.

[11] Kim D.-S., Lee S.-G., Jun S.-Y., Kyoung W. K., Ha T.-Y., Kim K.-K. (2008). Primary malignant mesothelioma developed in liver. *Hepato-Gastroenterology*.

[12] Gütgemann I., Standop J., Fischer H.-P. (2006). Primary intrahepatic malignant mesothelioma of epithelioid type. *Virchows Archiv*.

[13] Leonardou P., Semelka R. C., Kanematsu M., Braga L., Woosley J. T. (2003). Primary malignant mesothelioma of the liver: MR imaging findings. *Magnetic Resonance Imaging*.

[14] Imura J., Ichikawa K., Takeda J. (2002). Localized malignant mesothelioma of the epithelial type occurring as a primary hepatic neoplasm: a case report with review of the literature. *acta Pathologica, Microbiologica, et Immunologica Scandinavica*.

[15] Husain A. N., Colby T., Ordonez N. (2013). Guidelines for pathologic diagnosis of malignant mesothelioma: 2012 update of the consensus statement from the International Mesothelioma Interest Group. *Archives of Pathology and Laboratory Medicine*.

[16] Boffetta P. (2007). Epidemiology of peritoneal mesothelioma: a review. *Annals of Oncology*.

[17] Carbone M., Ly B. H., Dodson R. F. (2012). Malignant mesothelioma: facts, myths, and hypotheses. *Journal of Cellular Physiology*.

[18] Sekido Y. (2013). Molecular pathogenesis of malignant mesothelioma. *Carcinogenesis*.

[19] Mossman B. T., Shukla A., Heintz N. H., Verschraegen C. F., Thomas A., Hassan R. (2013). New insights into understanding the mechanisms, pathogenesis, and management of malignant mesotheliomas. *The American Journal of Pathology*.

[20] Matsuzaki H., Maeda M., Lee S. (2012). Asbestos-induced cellular and molecular alteration of immunocompetent cells and their relationship with chronic inflammation and carcinogenesis. *Journal of Biomedicine and Biotechnology*.

[21] Wei S. C., Zheng G. Q., Wang Z. G. (2013). The retrospective analysis of clinical data in 162 malignant peritoneum mesothelioma cases in Cangzhou. *Chinese Journal of Internal Medicine*.

[22] Sluis-Cremer G. K. (1991). Asbestos disease at low exposures after long residence times. *Annals of the New York Academy of Sciences*.

[23] Sluis-Cremer G. K., Liddell F. D. K., Logan W. P. D., Bezuidenhout B. N. (1992). The mortality of amphibole miners in South Africa, 1946–80. *British Journal of Industrial Medicine*.

[24] Boutin C., Dumortier P., Rey F., Villiat J. R., De Vuyst P. (1996). Black spots concentrate oncogenic asbestos fibers in the parietal pleura. *American Journal of Respiratory and Critical Care Medicine*.

[25] Craighead J. E. (2011). Epidemiology of mesothelioma and historical background. *Recent Results in Cancer Research*.

[26] Kindler H. L. (2013). Peritoneal mesothelioma: the site of origin matters. *American Society of Clinical Oncology Educational Book*.

[27] Wei S. C., Zheng G. Q., Wang Z. G. (2013). Retrospective analysis of the patients with peritoneal malignant mesothelioma in Cangzhou area. *Chinese Journal of Integrative Medicine*.

[28] Creaney J., Dick I. M., Dare H. (2013). Does CA125 binding to mesothelin impact the detection of malignant mesothelioma?. *Lung Cancer*.

[29] Ho M., Feng M., Fisher R. J., Rader C., Pastan I. (2011). A novel high-affinity human monoclonal antibody to mesothelin. *International Journal of Cancer*.

[30] Kebapci M., Vardareli E., Adapinar B., Acikalin M. (2003). CT findings and serum ca 125 levels in malignant peritoneal mesothelioma: report of 11 new cases and review of the literature. *European Radiology*.

[31] Zhang W., Wu X., Wu L., Zhao X. (2015). Advances in the diagnosis, treatment and prognosis of malignant pleural mesothelioma. *Annals of Translational Medicine*.

[32] Feong Y. J., Kim S., Kwak S. W. (2008). Neoplastic and non-neoplastic conditions of serosal membrane origin: CT findings. *Radiographics*.

[33] Comin C. E., Saieva C., Messerini L. (2007). h-Caldesmon, calretinin, estrogen receptor, and Ber-EP4: a useful combination of immunohistochemical markers for differentiating epithelioid peritoneal mesothelioma from serous papillary carcinoma of the ovary. *The American Journal of Surgical Pathology*.

[34] Takeshima Y., Amatya V. J., Kushitani K., Inai K. (2008). A useful antibody panel for differential diagnosis between peritoneal mesothelioma and ovarian serous carcinoma in Japanese cases. *American Journal of Clinical Pathology*.

[35] Husain A. N., Colby T. V., Ordóñez N. G. (2009). Guidelines for pathologic diagnosis of malignant mesothelioma: a consensus statement from the International Mesothelioma Interest Group. *Archives of Pathology and Laboratory Medicine*.

[36] Sterman D. H., Albelda S. M. (2005). Advances in the diagnosis, evaluation, and management of malignant pleural mesothelioma. *Respirology*.

[37] Yaziji H., Battifora H., Barry T. S. (2006). Evaluation of 12 antibodies for distinguishing epithelioid mesothelioma from adenocarcinoma: identification of a three-antibody immunohistochemical panel with maximal sensitivity and specificity. *Modern Pathology*.

[38] Ordóñez N. G. (2007). What are the current best immunohistochemical markers for the diagnosis of epithelioid mesothelioma? A review and update. *Human Pathology*.

[39] Taşkın S., Gümüş Y., Kiremitçi S., Kahraman K., Sertçelik A., Ortaç F. (2012). Malignant peritoneal mesothelioma presented as peritoneal adenocarcinoma or primary ovarian cancer: case series and review of the clinical and immunohistochemical features. *International Journal of Clinical and Experimental Pathology*.

[40] Sohrab S., Hinterthaner M., Stamatis G., Rödelsberger K., Woitowitz H.-J., Konietzko N. (2000). Das maligne pleuramesotheliom. *Deutsches Ärzteblatt*.

